# Alveolar Abscess

**Published:** 1881-04

**Authors:** L. D. Wilson


### THE DENTAL REGISTER.

Vol. XXXV.] APRIL, 1881. [No. 4.

### ALVEOLAR ABSCESS.

L. D. WILSON, DD. S.

Alveolar abscess is usually a result of dental periostitis, and some authors in treating of it, do not speak of it as a distinct affection, but simply as a termination of dental periostitis, when that affection is not checked in its earlier stages.

It is almost exclusively connected with teeth that have become devitalized; seldom attacking a tooth that has a live and healthy pulp.

The tooth itself is not always the original source of the disease. Some constitutional affection may be a predisposing cause. A local irritation kept up, at or near the root, as a bit of gold pushed through the apex of the root when filling it, and unperceived by the operator, may give rise to an abscess. Such causes will at first be very obscure, and the true cause not discovered until the tooth is extracted, or an opening made in some direction sufficient for the foreign body to make its escape. A malarticulation, undressed fillings, &c., &c., are indirect causes of abscess.

In abscess there is inflammation, a cavity formed, and an exudate poured out. The inflammation is the same as in other parts, except as modified by the anatomical difference of the part; or perhaps in some cases modified by the existence of some cachexia—as the scorbutic syphilitic, &c., &c. The membrane of the root becomes thicker, and shreddy in appearance; and as it becomes thicker, it becomes softer. This usually commences at the apex of the root, and extends toward the neck of the tooth. Pus is secreted by, or formed in this membrane; this makes pressure and causes severe pain—probably the most severe form of odontalgia. The inflammation in susceptible cases may extend over the surrounding tissues to an alarming extent, and be sufficiently severe to cause the sufferer to take to his bed. In a good constitution coagulated lymph forms about the root, sometimes there is only a very small quantity at the apex, and in the other cases it is quite abundant, filling up the space between the roots of a molar tooth, and sometimes to greater extent. It also varies in density; from being quite firm and resistant, to scarcely being able to support its own weight.

In persons of an enfeebled constitution, this lymph mass degenerates into pus, or may not be formed at all.

From the fact that this lymph mass is formed only in those of good systemic condition, it would seem that it is an attempt of nature to make a restoration of the part to health; but after treatment, the abscess does not heal by help of the lymph mass, and while most writers on alveolar abscess hint that it is nature's effort, they tell us the first thing to do is to break up the accumulated lymph. The character of the pus also varies—it is composed of degenerated plasm, white blood or pus corpuscles, bone in solution, &c., &c. Healthy or laudable pus is of a light straw or yellowish white color, of a creamy consistence, and sweetish to the taste. It may become dark colored, very acid and offensive, so much so as to excoriate healthy tissue with which it comes in contact.

The lymph mass accompanies healthy pus. The pus inclosed in the cavity increases, and forces a passage, more or less direct, in order to escape. Upon the escape of the pus, either by nature or by artificial means, the pain is lessened to a dull heavy pain, or may cease entirely.

The time occupied in the process varies in different cases, usually from two to ten days, it depends on the condition of the part prior to the disease, and the general condition of the patient. It proceeds more slowly, and greater harm results, in debilitated persons. Chronic abscesses may remain unnoticed for years, but may give rise to sympathetic pain.

The results of alveolar abscess are various. It sometimes occasions the loss of a tooth, and even of adjoining teeth, by necrosis of the process. Considerable damage may be done to the alveolar process without the knowledge of the individual in whose mouth it occurs. Fever and delirium sometimes, though rarely, accompany alveolar abscess, and has been known to cause the death of the patient from extensive necrosis.

The pus may reach the surface of the face from *any* tooth. The pus may escape in almost any direction, it may find exit in the fauces—into the maxillary sinus and escape through the nares. It may burrow along from a superior incisor, particularly from a lateral, between the periosteum and the bone, and find exit near the soft palate. It may find its way through the soft tissues of the neck, and open down as far as below the clavicle, particularly when from a third molar. It may discharge into the nares directly—in this case it is usually from a superior central incisor. The external opening of the fistula is, perhaps, more commonly at the gum opposite the apex of the tooth; or through the canal in the root. A third molar may be the cause of an abscess almost before it erupts, and these cases will often be obscure, especially when the opening is some distance from the tooth. Let us not be deceived as to the source of a fistulous opening on the face or elsewhere; we may sometimes discover its origin by following the course of the canal with an instrument. A dead tooth may remain in the mouth, but be apparently healthy; as in the case where the tubuli are almost obstructed, and do not take up coloring matter. Test the tooth to ascertain if dead by tapping it with an instrument in different directions, and by reflecting sun light on it from a mirror. Teeth containing fillings may have dead pulps. Persons of an inflammatory diathesis are the most prone to abscess.

The treatment varies with the particular case in hand. Formerly all abscessed teeth were extracted, but in these days of progress no such treatment should be tolerated, only in exceptional cases.

In a person of robust health, nature will occasionally make a complete cure.

When the pus begins to make pressure, the best treatment is to make an incision through the gum opposite the apex of the root, and with a drill-shaped instrument open through the alveolus and break up the lymph mass, if there be any, and inject in the cavity nitrate of silver in solution, or use the crystals; chloride of zinc has also been employed—a solution of two or three grains to the ounce of water. But perhaps better than the nitrate of silver or chloride of zinc is the tincture of iodine, aromatic sulphuric acid, and creosote and carbolic acid mixed. Apply one of the above remedies about once a day for two or three days, and after this a mild solution of creosote and tannin in alcohol applied on cotton or floss silk, daily. It is necessary to have good drainage; and sea tangle tent is a good article to slowly enlarge the opening, as it expands greatly when moistened.

In obstinate cases, we may sometimes extract, fill and replace the tooth in the socket. When the tooth is out we may plug the socket with a pleget of cotton, saturated with Pond's Extract of Hamamelis, or let the blood coagulate in the socket, and remove it just before replacing the tooth. Use great care in extracting, to lacerate the gums as little as possible.

If there be any objection to lancing the gum and perforating the alveolus, make a poultice of a roasted raisin or a fig, and apply to the gum opposite the root of the affected tooth.

Warm fomentations should not be employed on the face, as they are liable to cause the abscess to break on the face and leave a scar.

When they are liable to break on the cheek, use a compress of such nature as not to cause absorption; a sheet lead compress covered with some material saturated with some astringent solution, as alum water, and then make or induce an opening elsewhere.

Abscess may terminate in an osseous cyst, and the tissues over the bone remain normal. The pus accumulates, and instead of making an opening, expands the outer plate of the bone. It may be mistaken for a tumor, but is distinguished from it by its more rapid growth. It may be treated by the extraction of the tooth, or by opening the cyst and stuffing the cavity with lint saturated with some stimulant solution, as the tincture of iodine.

When the discharge takes place through the root of the tooth, there being an opening from the first, nothing is known of the abscess but from the taste of the pus. In such cases inject the solution through the canal of the tooth, or if this fail, make an opening through the gum and process, and if desired, fill the tooth permanently.

The most favorable place to treat alveolar abscess is in the upper jaw.

A discharge from about the neck of a tooth, caused by salivary calculus, could hardly be called a true abscess; and the treatment is to remove the cause, incise the gums, and make some stimulating application.

When the cases are chronic and resist local treatment, they will probably be found to be associated with some constitutional disturbance. Those cases in which there is mercurial poisoning, are the most obstinate. Rheumatism, gout and scrofula also induce to the chronic form. In case of mercurial poisoning the affected tooth may be so loose that extraction is advisable. Abscess in scrofulous children sometimes results in necrosis of the process, and abscess of the temporary teeth that do not yield readily to treatment, should be cured by extracting the tooth or teeth.

When an abscess is connected with some constitutional affection, or dependent upon it, treat that as a separate disease, together with the proper local treatment.

To consider the treatment of the several diseases which favor or induce abscess, is not intended for this paper.

